# Phase-defined complete sequencing of the HLA genes by next-generation sequencing

**DOI:** 10.1186/1471-2164-14-355

**Published:** 2013-05-28

**Authors:** Kazuyoshi Hosomichi, Timothy A Jinam, Shigeki Mitsunaga, Hirofumi Nakaoka, Ituro Inoue

**Affiliations:** 1 Division of Human Genetics, National Institute of Genetics, 1111 Yata, Mishima, Shizuoka 411-8540, Japan; 2 Department of Molecular Life Sciences, Tokai University School of Medicine, 143 Shimokasuya, Isehara, Kanagawa 259-1143, Japan

**Keywords:** HLA, Next generation sequencer

## Abstract

**Background:**

The human leukocyte antigen (HLA) region, the 3.8-Mb segment of the human genome at 6p21, has been associated with more than 100 different diseases, mostly autoimmune diseases. Due to the complex nature of HLA genes, there are difficulties in elucidating complete HLA gene sequences especially HLA gene haplotype structures by the conventional sequencing method. We propose a novel, accurate, and cost-effective method for generating phase-defined complete sequencing of HLA genes by using indexed multiplex next generation sequencing.

**Results:**

A total of 33 HLA homozygous samples, 11 HLA heterozygous samples, and 3 parents-child families were subjected to phase-defined HLA gene sequencing. We applied long-range PCR to amplify six HLA genes (*HLA-A, -C, -B, DRB1, -DQB1*, and *–DPB1*) followed by transposase-based library construction and multiplex sequencing with the MiSeq sequencer. Paired-end reads (2 × 250 bp) derived from the sequencer were aligned to the six HLA gene segments of UCSC hg19 allowing at most 80 bases mismatch. For HLA homozygous samples, the six amplicons of an individual were pooled and simultaneously sequenced and mapped as an individual-tagging method. The paired-end reads were aligned to corresponding genes of UCSC hg19 and unambiguous, continuous sequences were obtained. For HLA heterozygous samples, each amplicon was separately sequenced and mapped as a gene-tagging method. After alignments, we detected informative paired-end reads harboring SNVs on both forward and reverse reads that are used to separate two chromosomes and to generate two phase-defined sequences in an individual. Consequently, we were able to determine the phase-defined HLA gene sequences from promoter to 3′-UTR and assign up to 8-digit HLA allele numbers, regardless of whether the alleles are rare or novel. Parent–child trio-based sequencing validated our sequencing and phasing methods.

**Conclusions:**

Our protocol generated phased-defined sequences of the entire HLA genes, resulting in high resolution HLA typing and new allele detection.

## Background

The human leukocyte antigen (HLA) region on the chromosome 6p21 comprising six classical polymorphic HLA genes and at least 132 protein coding genes plays important roles in regulation of immune system as well as fundamental molecular and cellular processes [1]. The completion of a continuous 3.6 Mb of HLA genomic sequence together with annotation of 224 genes, was first reported by The MHC Sequencing Consortium in 1999 [2]. In addition, the MHC Haplotype Project conducted by the Sanger Institute provided genomic sequences and gene annotation of eight different HLA haplotypes, which were registered in the UCSC hg19 and NCBI GRCh37 reference assembly [3-5]. This 3.6 Mb segment occupies only 0.13% of the human genome but is associated with more than 100 different diseases, mostly autoimmune diseases such as type I diabetes, rheumatoid arthritis, psoriasis, and atopic asthma. Recently, HLA genes attracted special attentions, because specific alleles of HLA genes are strongly associated with drug hypersensitivity induced by specific drugs. For example, strong associations between carbamazepine-induced Stevens-Johnson syndrome (SJS) or toxic epidermal necrolysis (TEN) and HLA-B*15:02[6,7], abacavir-induced liver injury and HLA-B*57:01[8-11], and allopurinol-induced SJS or TEN and HLA-B*58:01[12] have been reported in various populations. For better understanding of disease causality and drug hypersensitivity, phase-defined complete HLA gene sequencing is required. Furthermore, complete HLA gene sequences are essential to minimize risk of graft versus host disease in hematopoietic transplantation because unknown determinants could be located around HLA genes.

Two methods of HLA genotyping, sequence specific oligonucleotide hybridization (SSO) and capillary sequencing with chain-termination reaction (Sanger sequencing or SBT), have been commonly applied in the past ten years. SSO requires the preparation of specific oligonucleotides corresponding to various genotypes in advance and potential difficulties may arise when new alleles are present. SBT or Sanger sequencing simultaneously sequences two chromosomes, thereby, phasing of the highly polymorphic HLA genes is very difficult *per se*. The common practice of SBT involves sequencing exons 2 and 3 of HLA Class I genes and exon 2 of HLA Class II genes. However, in some cases, different alleles share similar sequences across the sequenced region, leading to ambiguity in allele determination. Moreover, allele determination is generally based on sequence alignment to the IMGT/HLA database where there is an inherent limitation.

Rapid progress of sequencing technologies, so called next generation sequencing (NGS), resulted in revolutionary changes in medical genomics by providing massive sequencing data of human samples. Indeed, the 1,000 genomes project already reported novel variants including both rare and common types from population-scale sequencing [13]. Despite these progresses, complete sequence of HLA region could not be provided by the whole genome analysis because of the extraordinarily polymorphic and complex nature of the HLA region. Therefore, specific analytical procedures should be developed for completion of HLA sequencing and HLA haplotype determination. NGS technologies have potential advantages over Sanger method in sequencing HLA genes, i.e., sequence of single chromosome can be obtained at high throughput. Thus far, several high-throughput HLA typing methods using NGS have been developed [14-17]. One of those involved HLA class I typing by utilizing the 454 GS FLX Titanium sequencing platform with barcoding and multiplexing protocol, resulting in a 4-digit (fields 1 and 2 of HLA allele nomenclature) resolution with high accuracy in *HLA-A* (95.9%), *HLA-B* (99.4%), and *HLA-C* (94.4%) [16]. Recently, more comprehensive analyses of *HLA* typing using the Illumina platform were reported to demonstrate accurate genotyping via high coverage and extensive sequencing of the first seven exons of class I genes (*HLA-A, -B,* and *C*) and exons 2–5 of class II gene (*HLA-DRB1*) [17]. Also, cDNA amplicons of HLA genes were extensively sequenced [18,19] and these exon-centric analyses are successful in determining genotypes after consulting with the IMGT/HLA database to detect the closest HLA gene sequence. However, non-coding regions that may have impact on gene regulation [20,21], or mRNA splicing [22-24] are ignored. Most recently, 8-digit sequencing of HLA-genes is partially achieved using a combination of long-range PCR and Roche GS Junior sequencer and/or IonPGM sequencer [25]. In their study, the closest HLA gene sequence from the IMGT/HLA database was selected as the reference sequence for alignment and phasing, and subsequently they could construct consensus sequence to call HLA alleles. However, the phasing of single nucleotide variants (SNVs) separated at distances longer than the sequence reads, are dependent on the reference sequence because single read sequences of approximately 500 bp from GS Jr and 260 bp from IonPGM could not clarify phase ambiguities of those SNVs. In addition, if a target sequence is not registered in the database, it is not feasible to obtain complete sequences.

In the current study, we completely sequenced long-range PCR amplicons encompassing entire regions of each of the following HLA genes (*HLA-A, -C, -B, -DRB1, -DQB1,* and *-DPB1*). PCR amplicons were subjected to transposase-based library construction and multiplex sequencing with the MiSeq sequencer. Paired-end reads of 2 × 250 bp enables us to demonstrate phase-defined allele determination (also defined as HLA gene haplotype) for 33 HLA homozygous samples, 11 HLA heterozygous samples, and 3 parents-child families.

## Results

### PCR amplification of the HLA genes and library preparation

Genomic DNAs from 33 HLA homozygous cell lines, 11 heterozygous individuals, and 3 parents-child families were PCR amplified and subjected to HLA genes sequencing. We applied long-range PCR to amplify six HLA genes (*HLA-A, -C, -B, DRB1, -DQB1,* and *–DPB1*) that are known to be highly polymorphic. PCR primers were designed to anneal where known polymorphic sites were not observed according to the dbSNP build 135 database, and to amplify regions spanning the promoter to 3′UTR of the HLA genes (Additional file 1: Table S1). As shown in Additional file 2: Figure S1, specific amplification products of each gene were obtained; the PCR amplicon sizes of *HLA-A*, *-C*, *-B*, *-DRB1*, *-DQB1,* and *-DPB1* were 3,398 bp, 4,296 bp, 4,440 bp, 11,899 bp, 7,118 bp and 13,605 bp, respectively. Generally allelic imbalance and allele drop-out as a result of PCR is manifested by skewed allelic call in next generation HLA sequencing. Allelic imbalance of the PCR amplification for all the amplicons as judged by the sequencing results was 1:3.4 at the maximum ratio and 1:1.3 on average in heterozygous samples (Additional file 3: Table S2). In addition, extra sequences other than the target genes were observed. For example, in sequencing of *HLA-A* amplicon, sequences of *HLA-H* were observed as a low frequency extra amplification (<1.85%), even though we designed PCR primers at the specific sites of *HLA-A* and a single amplicon was observed by the gel electrophoresis. Although the *HLA-H* reads might generate false positive SNVs and disturb assembly results, the reads with low frequency SNVs, which is most likely regarded as false positive, were removed in our pipeline during the construction of the HLA gene haplotype. There is also a possibility that our primers may have amplified pseudogenes as an invisible amplification. However, we constructed only major combinations of SNVs for each specific loci, so minor amplifications did not disturb the sequencing results.

**Figure 1:**
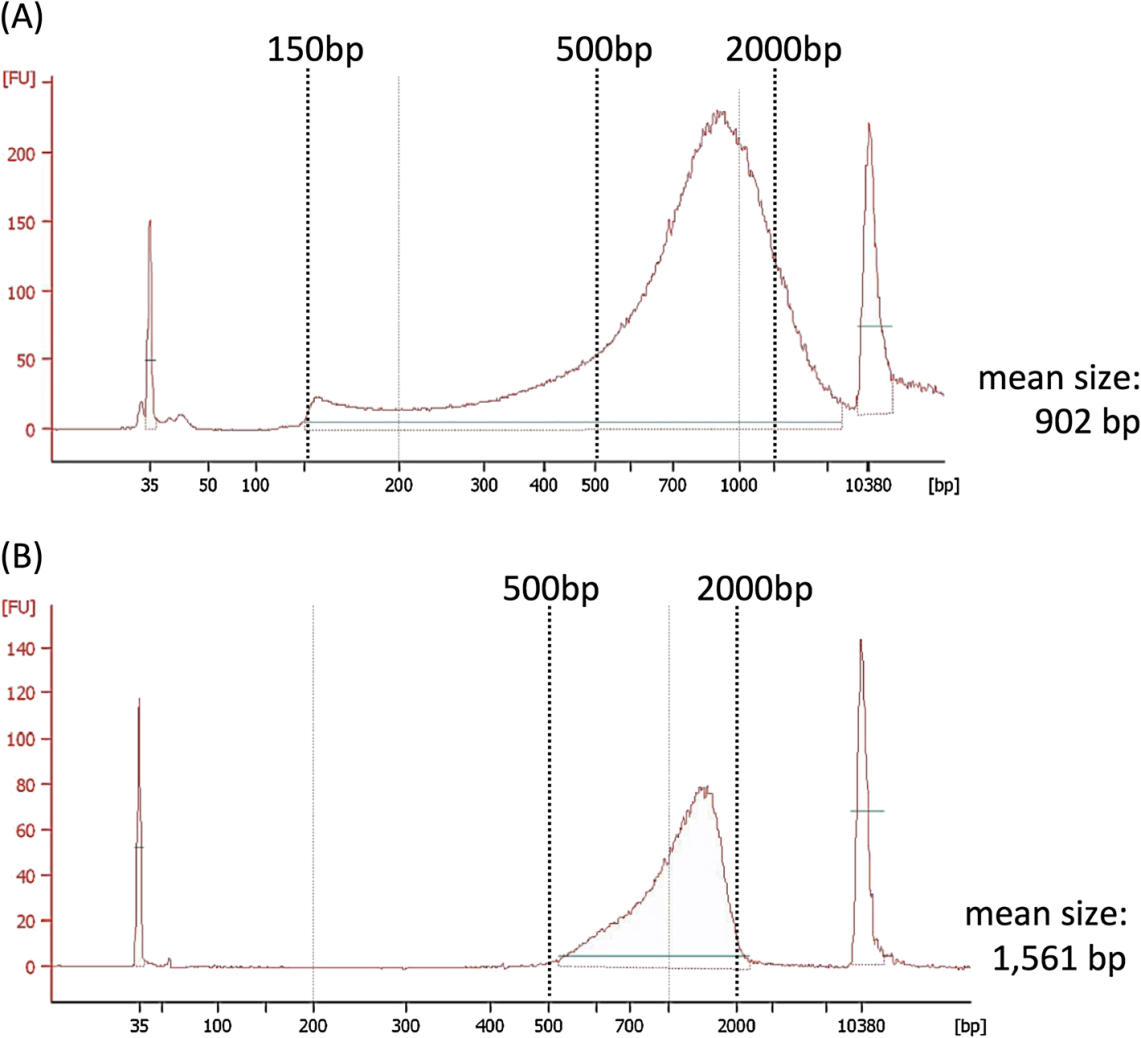
**Size selection of the Nextera DNA libraries by agarose gel size selection.** (**A**) Electropherogram of DNA library analyzed by 2100 Bioanalyzer. The library size of the Nextera DNA Sample Prep Kits was 150 bp to more than 10 kb (mean size: 902 bp). (**B**) Bioanalyzer electropherogram of a selected DNA library by cutting from the agarose gel. We selected large fragments with sizes ranging from 500 to 2,000 bp to remove short DNA fragments for effective HLA gene haplotype phasing. The size selection also determines an actual molar concentration for bridge PCR to generate clusters in flowcell, because DNA fragments with over 1.5 kb size are not efficiently amplified. The mean size of the selected fragments was 1,561 bp.

All of the PCR amplicons of six HLA genes from an individual were subjected to transposase-based library construction using the Nextera DNA Sample Prep Kit, which simultaneously adds adaptors needed to perform multiplex sequencing. For a practical reasons, we applied two different protocols for library preparation: In an individual-tagging method, all of the PCR amplicons of six HLA genes from an individual were pooled before being subjected to transposase treatment; In a gene-tagging method, each PCR amplicon was subjected to transposase-based library construction separately, whereby gene specific index was introduced. The Nextera kit is able to construct libraries of broad sizes (500 to 2,000 bp) by adding a gel purification step (Figure 1), and is able to attach indexes for up to 96 samples as described in the Methods.

### Individual-tagging method for complete sequencing of HLA homozygous samples

**Figure 2:**
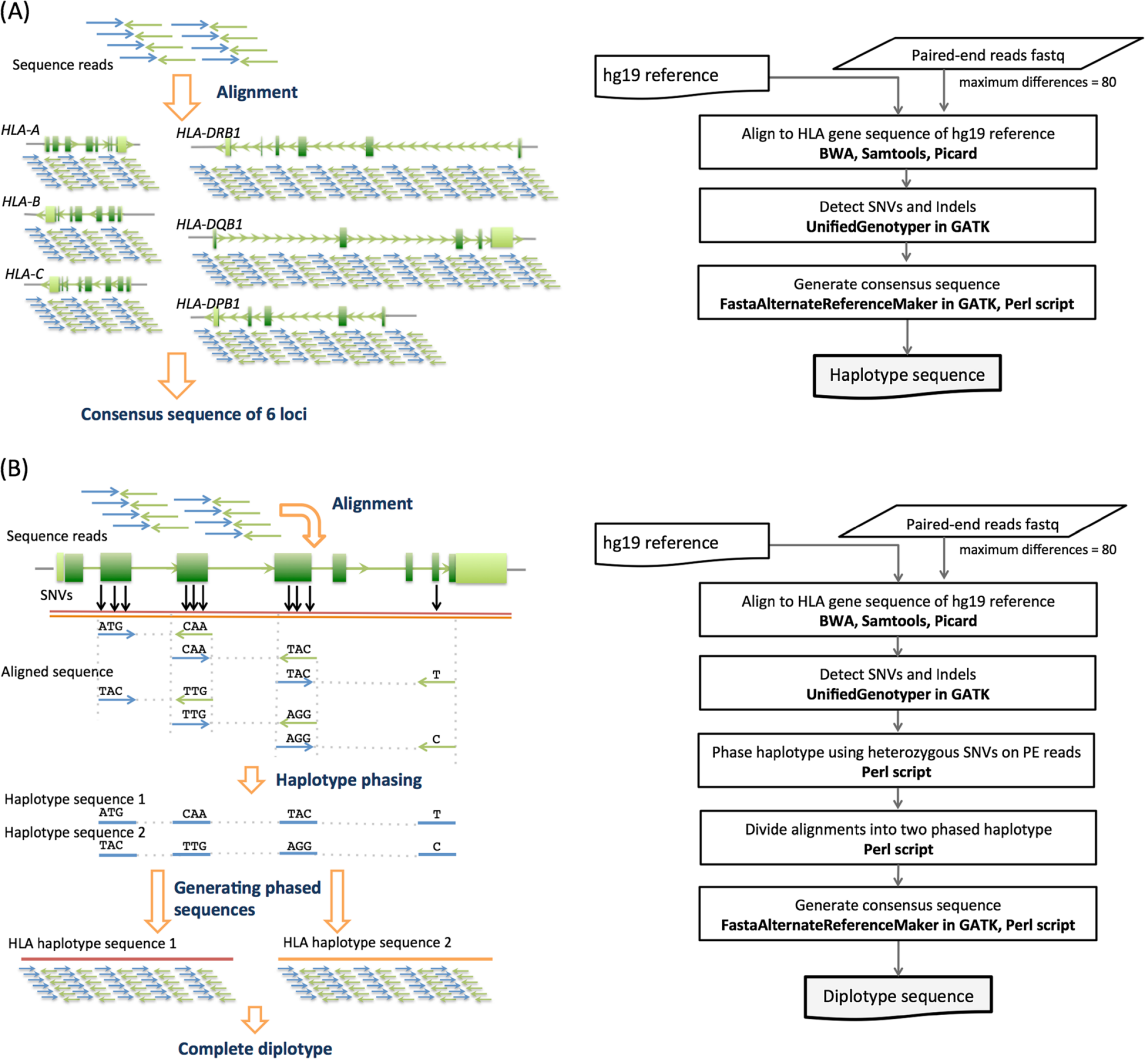
**Schematic workflow of the phase-defined HLA gene sequencing.** (**A**) The individual tagging method for HLA homozygous samples. The 2 × 250 bp paired-end reads of the pooled amplicons were aligned to six HLA gene sequences from the hg19 and the consensus sequences were determined. Most of the analytical tools shown here are standard use for genome sequence alignment and variant detection. For generating consensus sequence, we used original perl scripts to list variants and to construct HLA gene sequences. (**B**) The gene-tagging method for HLA heterozygous samples. The 2 × 250 bp paired-end reads of the each amplicon were aligned to the corresponding gene and six genes were separately analyzed to avoid mismapping. In the alignment step, 2 × 250 bp paired-end sequence reads were aligned to reference sequence using BWA and SAMtools. SNVs were detected by UnifiedGenotyper in GATK. Paired-end reads harboring SNVs in both forward and reverse reads were extracted to construct two phased HLA gene haplotype sequences using our original perl script. Finally, two HLA gene haplotype sequences from an individual were generated with phase-defined SNVs and Indels as HLA gene haplotypes.

The PCR amplicons of the six HLA genes of one individual were pooled (the individual-tagging method) and the Nextera-treated library was applied to sequencing analyses. Paired-end sequence reads (2 × 250 bp in length) from 33 HLA homozygous samples, mostly from established cell lines (Additional file 4: Table S3), were aligned to the HLA gene sequences of the reference sequence (UCSC hg19) allowing 80 bases mismatch in each 250-base-read derived from the MiSeq sequencer. Using the above parameters for alignment, on average 66.35% of paired-end reads were able to be aligned to the reference HLA gene sequence with an average depth of 157×. The tiling of overlapped paired-end reads results in a continuous sequence, which should directly represent the HLA gene haplotype in HLA homozygous samples (Figure 2A). Paired-end reads from a total of 198 amplicons were obtained (33 individuals; 6 amplicons per individual). Of those, HLA gene haplotype sequences could not be generated in 32 amplicons (3 for *HLA-A*, 2 for -*B*, 4 for -*DPB1*, 9 for -*DQB1*, 14 for -*DRB1*). This was due to the presence of long gaps caused by regions with extremely low depth, which prevented the generation of continuous gene sequences. All PCR amplicons were observed as a strong single band by the gel electrophoresis. However, the number of sequence reads from 32 amplicons were considerably low. This problem was caused by a biased amplification during library preparation of multiple sites using the individual-tagging method. We analyzed 166 amplicons to generate HLA gene sequences. Of those, we successfully generated 162 complete sequences which covered the entire span of the HLA gene (after both ends of amplicons nearby primer sites were trimmed) without any heterozygous sites. The remaining 4 sequences covered more than 99.7% of the entire HLA gene with up to 9 false heterozygous sites (Table 1). These heterozygous sites were later corrected as homozygous by an eyeball search using an alignment viewer and experimentally by the Sanger sequencing.

**Table 1:** Alignment result of the HLA gene sequence and genotype in the HLA homozygous cell lines

**Cell line**	**Gene**	**Allele**	**Average depth**	**False heterozygous SNVs**	Coverage^*^	**Closest HLA genotype**	**Note**
AKIBA	*A*	*24:02*	219.5	0	100	A*24:02:01:01	
	*C*	*12:02:02*	655.5	0	100	C*12:02:02	
	*B*	*52:01*	74.0	0	100	B*52:01:01:02	
	*DRB1*	*15:02*	346.9	0	100	DRB1*15:02:01	
	*DQB1*	*06:01:01*	33.2	0	100	DQB1*06:01:01	
	*DPB1*	*09:01*	604.9	0	100	DPB1*09:01	
AMAI	*A*	*68:02*	35.2	0	100	A*68:02:01:01	
	*C*	*04:01*	159.3	0	100	C*04:01:01:01	
	*B*	*53:01*	52.2	0	100	B*53:01:01	
	*DRB1*	*15:03*	92.6	0	100	DRB1*15:03:01	2bp (TG) deletion
	*DQB1*	*06:02*	133.6	0	100	DQB1*06:02:01	
	*DPB1*	*04:02*	94.9	0	100	DPB1*105:01	different from ECACC
ARBO	*A*	*03:01*	133.5	0	100	A*03:01:01:01	
	*C*	*06:02*	208.8	0	100	C*06:02:02:01	
	*B*	*58:02*	108.2	0	100	B*58:02	
	*DRB1*	*15:03*	43.4	-	86.6	not determined	
	*DQB1*	*06:02*	40.0	-	71.5	not determined	
	*DPB1*	*04:02*	100.6	0	99.2	not determined	
BM15	*A*	*01:01*	79.3	0	100	A*01:01:01:01	
	*C*	*07:01*	198.7	0	100	C*07:01:01	
	*B*	*49:01*	34.0	0	100	B*49:01:01	
	*DRB1*	*11:02*	90.9	0	98.3	not determined	
	*DQB1*	*03:01*	14.2	0	100	DQB1*03:01:01:01	2 intronic and 1 nonsynonymous novel variants
	*DPB1*	*03:01*	132.0	0	100	DPB1*03:01:01	24 intronic novel variants
BM16	*A*	*02:01*	28.6	0	100	A*02:01:01:01	
	*C*	*07:01*	249.0	0	100	C*07:01:01:01	
	*B*	*18:01*	183.3	0	100	B*18:01:01	3 intronic novel variants
	*DRB1*	*12:01*	55.4	0	100	DRB1*12:01:01	43 intronic novel variants
	*DQB1*	*03:01*	36.3	0	100	DQB1*03:01:01:01	1 intronic novel variant
	*DPB1*	*02:01:02*	58.3	0	100	DPB1*02:01:02	4bp (GGAA) insertion
BM21	*A*	*01:01*	61.0	0	100	A*01:01:01:01	
	*C*	*17:01*	233.0	0	100	C*17:01:01:02	
	*B*	*41:01*	176.3	0	100	B*41:01	
	*DRB1*	*11:01*	80.0	0	100	DRB1*11:01:01	
	*DQB1*	*03:01*	17.8	9^†^	99.7	DQB1*03:01:01:01	
	*DPB1*	*10:01*	103.8	0	100	DPB1*10:01	
BM92	*A*	*25:01:01*	24.1	0	100	A*25:01:01	
	*C*	*01:02*	458.0	0	100	C*01:02:01	
	*B*	*51:01:01*	218.0	0	100	B*51:01:01	
	*DRB1*	*04:04*	2.9	-	32.9	not determined	
	*DQB1*	*03:01*	1.1	-	0	not determined	
	*DPB1*	*04:02*	115.6	0	100	DPB1*04:02:01	
Boleth	*A*	*02:01:01:01*	25.7	1^†^	100	A*02:01:01:01	
	*C*	*03:04*	273.3	0	100	C*03:04:01:01	
	*B*	*15:01*	260.2	0	100	B*15:01:01:01	
	*DRB1*	*04:01:01*	27.1	0	98.4	not determined	
	*DQB1*	*03:02:01*	67.2	0	100	DQB1*03:02:01	
	*DPB1*	*04:01:01*	99.1	0	100	DPB1*04:01:01	
BSM	*A*	*02:01*	56.9	0	100	A*02:01:01:01	
	*C*	*03:04*	238.6	0	100	C*03:04:01:01	
	*B*	*15:01*	204.3	0	100	B*15:01:01:01	
	*DRB1*	*04:01*	18.3	0	98.4	not determined	
	*DQB1*	*03:02*	91.1	0	100	DQB1*03:02:01	
	*DPB1*	*02:01:02*	57.6	0	100	DPB1*02:01:02	
Carogero	*A*	*02:01*	28.2	0	100	A*02:01:01:01	
	*C*	*20:22*	22.8	0	100	C*02:02:02	
	*B*	*40:02*	6.1	-	91	not determined	
	*DRB1*	*-*	16.8	0	100	DRB1*16:01:02	
	*DQB1*	*-*	27.5	0	100	DQB1*05:02:01	
	*DPB1*	*04:01*	18.8	0	99.7	not determined	
COX	*A*	*01:01:01:01*	99.1	0	100	A*01:01:01:01	
	*C*	*07:01:01*	322.2	0	100	C*07:01:01	
	*B*	*08:01:01*	123.5	0	100	B*08:01:01	
	*DRB1*	*03:01*	284.9	0	100	DRB1*03:01:01	
	*DQB1*	*02:01*	367.3	0	100	DQB1*02:01:01	
	*DPB1*	*03:01*	270.5	0	100	DPB1*03:01:01	
DBB	*A*	*02:01*	454.2	0	100	A*02:01:01	
	*C*	*06:02*	636.8	0	100	C*06:02:01:01	
	*B*	*57:01*	142.5	0	100	B*57:01:01	
	*DRB1*	*07:01*	14.3	-	90.7	not determined	
	*DQB1*	*30:32*	1.9	-	70.8	not determined	
	*DPB1*	*04:01*	568.0	0	100	DPB1*04:01:01:01	
DHI	*A*	*31:01*	2.2	-	12.9	not determined	
	*C*	*12:03*	292.9	0	100	C*12:03:01:01	
	*B*	*38:01*	250.1	0	100	B*38:01:01	
	*DRB1*	*11:01*	57.2	0	100	DRB1*11:01:01	
	*DQB1*	*03:01*	140.1	0	100	DQB1*03:01:01:03	
	*DPB1*	*04:01*	302.2	0	100	DPB1*04:01:01:01	
DKB	*A*	*24:02*	358.6	0	100	A*24:02:01	1 intronic novel variant
	*C*	*03:04*	502.8	0	100	C*03:04:01:01	
	*B*	*40:01*	634.1	0	100	B*40:01:02	
	*DRB1*	*90:12*	61.4	-	65	not determined	
	*DQB1*	*30:32*	310.1	0	100	DQB1*03:03:02:02	
	*DPB1*	*04:01*	259.2	0	100	DPB1*04:01:01:01	
HARA	*A*	*24:02:01:01*	122.6	0	100	A*30:01:01	different from ECACC
	*C*	*12:02*	214.3	0	100	C*12:02:02	
	*B*	*52:01:01*	189.1	0	100	B*52:01:01:02	
	*DRB1*	*15:02:01*	4.5	0	97.5	not determined	
	*DQB1*	*06:01*	124.6	0	100	DQB1*06:01:01	
	*DPB1*	*09:01*	52.9	-	97.6	not determined	
HHK	*A*	*03:01*	265.9	0	100	A*03:01:01:01	
	*C*	*07:02*	84.6	0	100	C*07:02:01:03	
	*B*	*07:02*	11.5	0	100	B*07:02:01	
	*DRB1*	*13:01*	47.7	0	100	DRB1*13:01:01	
	*DQB1*	*06:03*	103.3	0	100	DQB1*06:03:01	
	*DPB1*	*04:01*	65.1	0	100	DPB1*04:01:01:01	
HOKKAIDO	*A*	*-*	8.0	0	100	A*24:02:01:01	
	*C*	*-*	42.8	0	100	C*03:04:01:02	
	*B*	*-*	99.0	0	100	B*54:01:01	
	*DRB1*	*04:05*	8.2	-	11.6	not determined	
	*DQB1*	*04:01*	48.4	-	57.4	not determined	
	*DPB1*	*05:01*	70.4	0	100	DPB1*05:01:01	
JBUSH	*A*	*32:01*	10.9	1^†^	100	A*32:01:01	
	*C*	*12:03*	128.8	0	100	C*12:03:01:01	
	*B*	*38:01*	298.3	0	100	B*38:01:01	
	*DRB1*	*11:01*	23.7	0	100	DRB1*11:01:03	
	*DQB1*	*03:01*	80.1	0	100	DQB1*03:01:01	
	*DPB1*	*04:01*	86.7	0	100	DPB1*04:01:01:01	
JEST	*A*	*02:01*	11.3	0	100	A*02:01:01	
	*C*	*01:02*	80.3	0	100	C*01:02:01	
	*B*	*27:05:02*	76.8	0	100	B*27:05:02	1 intronic novel variant
	*DRB1*	*01:01*	16.3	0	100	DRB1*01:01:01	
	*DQB1*	*05:01*	87.5	0	100	DQB1*05:01:01	1 intronic novel variant
	*DPB1*	*04:01*	69.1	0	100	DPB1*04:01:01:01	
K265	*A*	*-*	127.1	0	100	A*03:01:01:01	
	*C*	*12:02*	163.7	0	100	C*07:02:01:03	
	*B*	*-*	54.3	0	100	B*07:02:01	
	*DRB1*	*15:02*	92.7	0	100	DRB1*15:01:01	
	*DQB1*	*-*	257.5	0	100	DQB1*06:02:01	
	*DPB1*	*-*	107.8	0	100	DPB1*04:01:01:01	
LBUF	*A*	*30:01:01*	26.9	0	100	A*30:01:01	
	*C*	*06:02*	326.3	0	100	C*06:02:01:01	
	*B*	*13:02:01*	249.1	0	100	B*13:02:01	
	*DRB1*	*07:01:01*	16.6	0	100	DRB1*07:01:02	
	*DQB1*	*02:02*	2.9	-	18.9	not determined	
	*DPB1*	*17:01*	177.9	0	100	DPB1*17:01	
LKT3	*A*	*24:02*	86.9	0	100	A*24:02:01	
	*C*	*01:02*	213.3	0	100	C*01:02:01	
	*B*	*54:01*	48.7	0	100	B*54:01:01	
	*DRB1*	*08:01*	23.4	-	55.4	not determined	
	*DQB1*	*04:01*	1.1	-	0	not determined	
	*DPB1*	*05:01*	227.8	0	100	DPB1*05:01:01	
MADULA	*A*	*02:01*	86.8	0	100	A*02:01:01	
	*C*	*03:04*	206.9	0	100	C*03:04:01	
	*B*	*40:01*	223.8	0	100	B*40:01:02	
	*DRB1*	*08:01*	42.6	0	100	DRB1*08:01:03	
	*DQB1*	*04:02*	123.9	-	86.3	not determined	
	*DPB1*	*04:01*	215.6	0	100	DPB1*04:01:01	
PITOUT	*A*	*29:02*	91.5	0	100	A*29:02:01	
	*C*	*16:01*	51.8	0	100	C*16:01:01	
	*B*	*44:03:01*	74.6	0	99.4	not determined	
	*DRB1*	*07:01*	3.2		40.4	not determined	
	*DQB1*	*02:01*	38.3	0	100	DQB1*02:02	different from ECACC
	*DPB1*	*04:01*	340.7	0	100	DPB1*04:01:01	
RMAL	*A*	*02:04*	75.8	0	100	A*02:04	
	*C*	*15:02*	68.6	0	100	C*15:02:01	
	*B*	*51:01*	21.9	0	100	B*51:01:01	
	*DRB1*	*16:02*	63.5	0	100	DRB1*16:02:02	3 intronic novel variants
	*DQB1*	*03:01*	16.2	0	100	DQB1*03:01:01	
	*DPB1*	*04:02*	66.8	0	100	DPB1*04:02:01	
SAVC	*A*	*03:01*	99.4	0	100	A*03:01:01:01	
	*C*	*07:02*	140.3	0	100	C*07:02:01	
	*B*	*07:02*	45.3	0	100	B*07:02:01	
	*DRB1*	*04:01*	2.4	-	50.3	not determined	
	*DQB1*	*03:02*	1.2	-	12.9	not determined	
	*DPB1*	*10:01*	129.2	0	100	DPB1*10:01	
SRACH	*A*	*31:01*	14.5	-	1.5	not determined	
	*C*	*01:02*	388.3	0	100	C*01:02:01	
	*B*	*15:01*	688.5	0	100	B*15:01:0101	
	*DRB1*	*08:02:01*	61.5	0	100	DRB1*08:02:01	
	*DQB1*	*04:02*	174.0	-	91	not determined	
	*DPB1*	*04:02*	207.0	0	100	DPB1*04:02:01	
T182	*A*	*24:02*	12.4	0	100	A*24:02:01:01	
	*C*	*12:02:02*	113.5	0	100	C*12:02:02	
	*B*	*52:01*	106.0	0	100	B*52:01:01:02	
	*DRB1*	*15:02*	4.9	-	0	not determined	
	*DQB1*	*06:01*	72.2	0	100	DQB1*06:01:01	
	*DPB1*	*09:01*	69.2	0	98.3	not determined	
TAB089	*A*	*02:07*	13.3	0	98.3	not determined	
	*C*	*01:02*	101.9	0	100	C*01:02:01	
	*B*	*46:01*	36.9	0	100	B*46:01:01	
	*DRB1*	*02:02*	2.7	-	91.1	not determined	
	*DQB1*	*06:01*	49.1	1^†^	100	DQB1*06:01:01	
	*DPB1*	*02:02*	39.2	0	100	DPB1*02:02	9 intronic novel variants
TOK	*A*	*24:02*	286.2	0	100	A*24:02:01:01	
	*C*	*12:02:02*	241.5	0	100	C*12:02:02	
	*B*	*52:01*	127.9	0	100	B*52:01:01:02	
	*DRB1*	*15:02*	163.7	0	100	DRB1*15:02:01	
	*DQB1*	*06:01*	243.1	0	100	DQB1*06:01:01	
	*DPB1*	*09:01*	175.2	0	100	DPB1*09:01	
VAVY	*A*	*01:01*	36.1	0	100	A*01:01:01:01	
	*C*	*07:01*	141.6	0	100	C*07:01:01	
	*B*	*08:01*	535.1	0	100	B*08:01:01	
	*DRB1*	*03:01*	43.4	0	100	DRB1*03:01:01	
	*DQB1*	*02:01*	126.4	0	100	DQB1*02:01:01:01	
	*DPB1*	*01:01*	57.0	0	100	DPB1*01:01:01	
WT100	*A*	*11:01*	66.4	0	100	A*11:01:01	
	*C*	*04:01*	112.9	0	100	C*04:01:01:02	
	*B*	*35:01*	15.9	0	100	B*35:01:01	
	*DRB1*	01:01	143.5	0	100	DRB1*01:01:01	
	*DQB1*	*05:01*	136.4	0	100	DQB1*05:01:01	1 intronic novel variant
	*DPB1*	*01:01*	122.0	0	100	DPB1*01:01:01	
WT47	*A*	*32:01*	56.5	0	100	A*32:01:01	
	*C*	*05:01*	192.5	0	100	C*05:01:01	
	*B*	*44:02*	47.8	0	100	B*44:02:01	
	*DRB1*	*13:02*	116.1	0	100	DRB1*13:02:01	4 intronic novel variants
	*DQB1*	*06:04*	807.8	0	100	DQB1*06:04:01	
	*DPB1*	*16:01*	172.2	0	100	DPB1*16:01	

^*^ Percentage of the HLA gene covered by reads that passed the Q20 threshold for nucleotide quality.

^†^ Confirmed by sanger sequence.

HLA allele calls for these sequences were obtained by referring to the IMGT/HLA database, where mostly complete gene sequences were searched (Table 1). Of the 166 completely homozygous sequences, 152 sequences were identical to the complete gene sequences recorded in the IMGT/HLA database. 14 were found to be novel sequences, although they are quite similar to the Database sequences. Most of the novel variants were observed in the intronic region. Only one novel non-synonymous variant were detected in *DQB1* of sample BM15 (Table 1). Ultimately, we could obtain complete sequences without misalignment for majority of amplicons of the pooled PCR amplicons by the individual-tagging method for homozygous samples.

We attempted to apply the individual-tagging method to the 11 HLA heterozygous samples. However, we could not obtain phase-defined sequences, probably due to mismapping of paired-end reads, as evidenced by the high detection rate of the variants. Therefore, we applied the gene-tagging method for HLA heterozygous samples.

### Gene-tagging method for complete sequencing of HLA heterozygous samples

For HLA heterozygous samples, each PCR amplicon was separately subjected to the transposase-based library construction for sequencing as the gene-tagging method. We successfully obtained sequences of 6 genes in 11 HLA heterozygous individuals, resulting in a total of 66 amplicons. Paired-end reads (2 × 250 bp) from an amplicon were aligned to the respective HLA gene of the hg19 reference, allowing maximum 80 mismatches per read. On average, 73.1% of all reads could be successfully mapped to the reference sequence for all 66 amplicons. Overall, the average depth for the 66 amplicons ranged from 146× to 6,678×, with a mean of 2,281× (Table 2). In general, HLA class I genes tend to have higher average coverage (3,405×) compared to HLA class II genes (1,157×), which may be due to the larger amplicon sizes for HLA class II genes.

**Table 2:** Sequence quality and closest HLA allele designation for heterozygous samples

**Sample**	**Gene**	**Average depth**	**Coverage**^**¶**^**(allele 1/allele 2)**	**Luminex genotype**	**Closest HLA genotype**
E1	*A*	4306.6	97.1/100	*02:01/31:01*	*02:01:01:01/31:01:02*
	*C*	6668.5	100/99.6	*03:03/03:04*	*03:03:01/03:04:01:01*
	*B*	5250.9	100/100	*15:11/40:01*	15:11:01^*^/40:01:02
	*DRB1*	272.2	99.7/95.6	*04:05/09:01*	04:05:01/09:01:02^*^
	*DQB1*	3322.3	100/99.5	*03:03/04:01*	*03:03:02:02/04:01:01*
	*DPB1*	780.8	95.5/100	*02:02/05:01*	02:02/05:01:01^*^
E8	*A*	4303.7	100^†^	*24:02/24:02*	*24:02:01:01/24:02:01:01*
	*C*	3904.7	100/100	*12:02/14:02*	*12:02:02/14:02:01*
	*B*	2829.4	99.7/100	*51:01/52:01*	*51:01:01/52:01:01:01*
	*DRB1*	612.3	100/100	*01:01/15:02*	*01:01:01/15:02:01*
	*DQB1*	3132.6	100/100	*05:01/06:01*	*05:01:01:02/06:01:01*
	*DPB1*	588.7	100/100	*04:02/09:01*	*04:02:01:02/09:01*
E11	*A*	3880.2	100/100	*24:02/31:01*	*24:02:01:01/31:01:02*
	*C*	3695.0	100/100	*01:02/07:04*	*01:02:01/07:04:01*
	*B*	4490.7	100/100	*15:18/54:01*	15:18:01^*^/54:01:01
	*DRB1*	501.8	78.3/99.2	*04:01/04:05*	*04:01:01/04:05:01*
	*DQB1*	3751.8	100/100	*03:01/04:01*	03:01:01:01/04:01:01^*^
	*DPB1*	748.2	100/100	*02:01/05:01*	02:01:02^*^/05:01:01^*^
E17	*A*	808.4	100/100	*02:07/24:02*	*02:07:01/24:02:01:01*
	*C*	1708.7	100/100	*01:02/12:02*	*01:02:01/12:02:02*
	*B*	2507.7	100/100	*46:01/52:01*	*46:01:01/52:01:01:02*
	*DRB1*	2080.3	100/100	*08:03/15:02*	*08:03:02/15:02:01*
	*DQB1*	1638.1	100^†^	*06:01/06:01*	*06:01:01/06:01:01*
	*DPB1*	1649.6	97.9/98.4	*05:01/09:01*	05:01:01^*^/09:01
E25	*A*	1042.7	100/100	*02:06/24:02*	*02:06:01/24:02:01:01*
	*C*	2150.0	100/100	*07:02/12:02*	*07:02:01:03/12:02:02*
	*B*	4871.7	100/100	*07:02/52:01*	*07:02:01/52:01:01:02*
	*DRB1*	460.3	100/32.8	*01:01/09:01*	01:01:01^*^/09:01:02^*^
	*DQB1*	1396.3	100/100	*03:03/05:01*	03:03:02:02/05:01:01:02^*^
	*DPB1*	2289.0	99.6/100	*02:02/04:02*	02:02^*^/04:02:01:01^*^
E28	*A*	1645.3	97.2/100	*26:01/33:03*	26:01:01/33:03:01^*^
	*C*	824.8	99.7^†^	*03:04/03:04*	*03:04:01:02/03:04:01:02*
	*B*	2799.6	100/100	*40:02/40:06*	40:02:01^*^/40:06:01:01
	*DRB1*	236.5	100^†^	*09:01/09:01*	09:01:02^*^/09:01:02^*^
	*DQB1*	817.5	100^†^	*03:03/03:03*	*03:03:02:02/03:03:02:02*
	*DPB1*	343.0	100^†^	*02:01/02:01*	*02:01:02/02:01:02*
E30	*A*	1711.4	100/100	*24:02/33:03*	24:02:01:01/33:03:01^*^
	*C*	2670.8	100/100	*01:02/14:03*	01:02:01^*^/14:03
	*B*	1899.1	100/100	*44:03/55:02*	*44:03:01/55:02:01*
	*DRB1*	399.4	17.1/99.8	*09:01/13:02*	09:01:02^*^/13:02:01^*^
	*DQB1*	773.1	100/97.8	*03:03/06:04*	03:03:02:02/06:09^*^
	*DPB1*	460.4	100/100	*04:01/04:01*	*04:01:01/04:01:01*
M13	*A*	3689.7	97.2/100	*11:01/24:02*	*11:01:01/24:02:01:01*
	*C*	3386.3	100/100	*01:02/04:01*	*01:02:01/04:01:01:01*
	*B*	3508.5	100/100	*15:01/59:01*	*15:01:01:01/59:01:01:01*
	*DRB1*	1029.3	100/99.5	*04:05/09:01*	04:05:01/09:01:02^*^
	*DQB1*	577.6	100/100	*03:03/04:01*	*03:03:02:02/4:01:01*
	*DPB1*	1981.8	100/100	*04:02/05:01*	04:02:01:02/05:01:01^*^
M14	*A*	1690.3	100/100	*31:01/33:03*	*31:01:02/33:03:01*
	*C*	4353.2	100/100	*04:01/06:02*	*04:01:01:01/06:02:01:01*
	*B*	4733.5	100/100	*15:01/57:01*	*15:01:01:01/57:01:01*
	*DRB1*	446.2	100/99.5	*15:01/07:01*	15:01:01:02*/07:01:01
	*DQB1*	763.0	98.8/98.9	*03:03/06:02*	*03:03:02:01/06:02:01*
	*DPB1*	1209.5	99.8/100	*09:01/04:02*	09:01/04:02:01:02^*^
M15	*A*	1153.2	100/100	*31:01/33:03*	*31:01:02/33:03:01*
	*C*	1448.1	99.7/99.8	*14:02/14:03*	*14:02:01/14:03*
	*B*	3312.9	100/100	*44:03/51:01*	*44:03:01/51:01:01*
	*DRB1*	463.4	100^†^	*04:05/04:05*	*04:05:01/04:05:01*
	*DQB1*	280.9	100^†^	*04:01/04:01*	04:01:01^*^/04:01:01^*^
	*DPB1*	2932.9	99.5/100	*02:01/03:01*	*02:01:02/03:01:01*
M20	*A*	7631.1	100/100	*02:06/11:01*	*02:06:01/11:01:01*
	*C*	7675.7	100/100	*03:03/08:01*	*03:03:01/08:01:01*
	*B*	7566.7	100/100	*15:02/35:01*	15:02:01/35:01:01:01^*^
	*DRB1*	4746.2	100^†^	*15:01/15:01*	*15:01:01:01/15:01:01:01*
	*DQB1*	6952.9	100/100	*06:01/06:02*	*06:01:01/06:02:01*
	*DPB1*	4631.3	100/99.6	*05:01/02:02*	*05:01:02/02:02*

^¶^ Percentage of the HLA gene covered by reads that passed the Q20 threshold for nucleotide quality.

^*^Mismatched bases with recorded sequences in IMGT/HLA database observed.

^†^ Homozygous HLA allele.

First, we tried to detect informative paired-end reads harboring SNVs on both forward and reverse reads; this information is used to separate two chromosomes and to determine the two phase-defined HLA gene sequences. Taking advantage of highly polymorphic nature of the HLA genes, wide-ranged library size, and deep sequencing, it becomes possible to phase sequence reads on a chromosome and tile phased reads to generate HLA gene haplotype sequences (Figure 2B). After alignment to the respective HLA gene reference sequence (hg19), two HLA gene haplotype sequences based on phase-defined SNVs were generated (Figure 2B). Overall, we were able to obtain 132 phase-defined sequences (66 amplicons; 2 chromosomes each). Of those, we successfully generated 103 complete haploid sequences which covered the entire span of the HLA gene (after both ends of amplicons nearby primer sites were trimmed). The remaining 26 haploid sequences covered more than 95% of the entire HLA gene whereas another three had less than 95% coverage (Table 2). The incomplete coverage of the HLA genes was due to remaining unphased regions, which may include large gaps. Most notably, our phase-defined sequencing does not refer to the IMGT/HLA database in order to generate HLA gene sequences unlike HLA typing methods using NGSs reported thus far.

After obtaining phase-defined HLA gene sequences for 132 haploid sequences, we tried to designate HLA allele numbers to these sequences by searching for known allele sequences in the IMGT/HLA database. First, we used the phased HLA gene haplotype sequences that spanned all of the intronic and exonic regions of the HLA gene, as a query against complete gene sequences in the database. Because sequencing efforts of the HLA region tend to focus on limited exons, the number of available complete gene sequences in the database tend to be lacking compared to coding region sequences. If we did not get any hit in the complete HLA gene sequence database, we extracted exon sequences from our complete, phased HLA gene haplotype sequences to obtain HLA coding sequences and searched them for known cDNA sequences in the database. Overall, we were able to determine the closest HLA allele number for all of our HLA gene haplotype sequences by searching for the database (Table 2). Of those, 104 alleles were obtained by searching for the complete gene sequences and another 28 obtained by searching the cDNA database. We managed to obtain perfect match with the database in 100 HLA gene haplotypes of the heterozygous samples, resulting in 6 to 8 digit HLA allele resolution. Another 32 HLA gene haplotypes recorded 1 to 5 bp mismatches with known sequences in the database, where 17 HLA gene haplotypes had mismatches in the exonic region and 15 HLA gene haplotypes had mismatches in the intronic region. These mismatches may be due to new alleles, reflecting deficiencies of the database, or be due to our sequencing error. In conclusion, we could successfully determine the HLA allele numbers for all of HLA sequences.

### Parent/child trios for the confirmation of the HLA gene sequences

**Figure 3:**
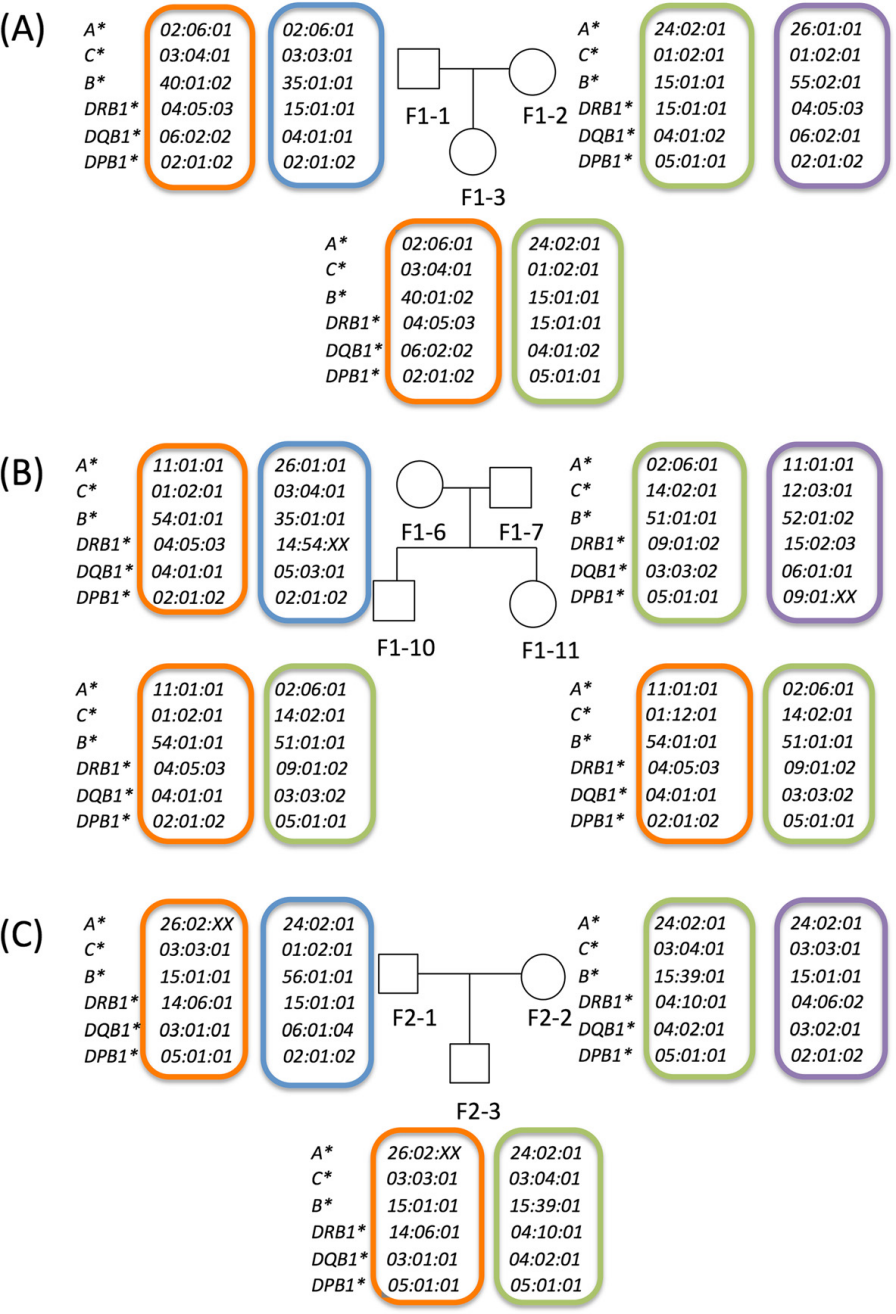
**The HLA alleles and HLA haplotypes in two trio (A and C) one quartet (B) families.** Each individual in child-parents families was sequenced as described. Each HLA gene call was consistent with the hereditary pattern. HLA allele was inferred by the IMG/HLA database and shared between parents and child(ren) with consistent pattern and without recombination.

Phase-defined HLA gene haplotype structure is classically confirmed by the cloning and Sanger sequencing method, because it is not feasible to define the phase by using the Sanger sequencing only. Because the cloning and sequencing method is laborious, inefficient, and impractical for sequencing massive samples, we took a genetic approach applying parents/child trios to define the phase of the gene sequences and also to confirm the fidelity of our results. Individuals from three trio (parents and child pair) families were all subjected to the HLA gene sequencing protocol as previously described using the gene-tagging method. All the phase-defined sequencing data of the HLA genes were consistent with the SSO genotyping data (data not shown) and the hereditary pattern confirming the validity of our phase-defined sequencing and analytical pipeline (Figure 3). HLA gene haplotype structure is inferred by the family structure and recombination event in three trio families was not observed.

## Discussion

The primary goal of our current study is to determine phase-defined complete HLA-gene sequencing without ambiguity. The six highly polymorphic HLA genes (*HLA-A, -C, -B, -DRB1, -DQB1,* and *-DPB1*) were amplified by long-range PCR and the PCR amplicons covering full sequences of the genes were subjected to the MiSeq sequencer via the transposase-based library preparation. The derived paired-end reads (2 × 250 bp) from the MiSeq sequencer were analyzed by the one step alignments to the UCSC hg19 to obtain phase-defined complete sequences.

Thus far, several methods to sequence HLA genes with next generation sequencers were reported. These methods are based on PCR-amplification of specific regions of HLA genes followed by NGS, mostly using the 454 GS FLX Titanium sequencing platform [14-16]. Derived sequence reads were usually mapped to a reference library of HLA gene sequences such as the IMGT-HLA database in the alignment step. Then, the closest sequences are selected as the reference sequences for alignment. Because only a limited number of full sequences of the HLA genes is registered in the database, derived complete gene sequences are hardly mapped. Accordingly, most studies only map exon sequences on cDNA references from the IMGT-HLA database. For example, because exons 2 and 3 of class I genes and exon 2 of class II genes are highly polymorphic and critical for antigen presentation, several studies practically focused on sequencing these exons [14,16]. Recent publications showed more extensive sequencing targeting all exons by PCR or cDNA amplification demonstrating complete sequencing of the coding regions [18,19]. If exons are separately sequenced, phased HLA gene sequence (or HLA gene haplotype) can not be defined, indeed, the HLA gene haplotype was inferred by the IMGT-HLA database. The complete cDNA sequencing can automatically define the HLA gene haplotype structures of the exons without inference [18,19], however, preparation of cDNA is not practical, especially considering the clinical application. The exon-centric analyses are efficient in data-analyses, however, two fundamental shortcomings need to be reminded: First, it has been reported that expression level of HLA genes is associated with disease phenotype [20,21], thus genetic variants in regulatory element such as promoter or even introns need to be extensively analyzed. In fact, a variant of intron 2 of *HLA-A* was reported as causality of low expression of the HLA gene, furthermore, variants in intron 2 of *HLA-A*, introns 2 and 4 of *HLA-B* cause null expression by cryptic splicing activation [22-24]. Thus, promoter/enhancer, intron, and 3′-UTR variants should not be ignored for comprehensive HLA typing. Second, because of high linkage disequilibrium in the HLA region, true causalities of diseases and drug adverse effects including regulatory variants can not be identified unless phase-defined complete gene sequence and HLA haplotype structure are achieved. Because of that, Shiina *et al.* established the sequence-based typing method for the 8 HLA genes (*HLA-A, -B, -C, -DRB1, -DQA1, -DQB1, -DPA1,* and *-DPB1*) sequencing up to 8-digit resolution [25]. But their protocol basically relies on the IMGT-HLA database and HLA gene haplotype determination is based on inference, thus phase-defined HLA gene haplotype structure of novel sequence could hardly be achieved.

We report here an analytical pipeline for determining phase-defined complete sequencing of the six HLA genes. The long-range PCR products of HLA genes spanning from promoter to 3′-UTRs were prepared and sequenced by the MiSeq sequencer via the transposase-based library preparation. We applied only one reference sequence, UCSC hg19, for alignment, which not only simplifies the analytical step but also can accommodate all the samples. This is the major advantage using the proposed protocol because novel allele could be determined by our sequencing pipeline. Also we prepared a large and broad sized library (500–2,000 bp) using the Nextera kit, which can generate paired-end reads of a variety of sizes (Figure 1), which facilitates our phase-defined sequencing method. Overall, we determined 162 HLA gene haplotype sequences from homozygous cell lines, 132 HLA gene haplotype sequences from heterozygous samples and 60 HLA gene haplotype sequences from trio/quartet from 3 families. We applied two different procedures to prepare DNA library for MiSeq sequencing; the individual-tagging and gene-tagging methods. The Nextera DNA Sample Preparation Kit enables tagging of up to 96 libraries with unique dual indexes, which are added via PCR primers during the final amplification step. In the individual-tagging method, 576 amplicons derived from 6 HLA genes of 96 samples can be analyzed in one run, whereas 96 amplicons are analyzed using the gene-tagging method. We are aware that the current protocol for HLA heterozygous samples with the gene-tagging method would be low throughput and costly compared with the individual-tagging method, although providing accurate genotyping. Evidently, there is much room to be improved. We are in a stage to develop a new mapping method for the complete sequencing of the HLA heterozygous samples by the individual-tagging method.

## Conclusions

We established the high throughput, high resolution, and high fidelity HLA genotyping using transposase-based library construction and multiplex sequencing with MiSeq sequencer. Using the transposase-based library preparation method, it becomes feasible to construct multi-libraries (up to 96 libraries) with dual index at once for only 90 minutes. These could be greatly advantageous for clinical (diagnostic) application that requires a user-friendly and cost-effective protocol, with high throughput and accuracy. In conclusion, we are able to determine the phase-defined entire HLA gene sequences, regardless whether the alleles are rare or novel.

## Methods

### DNA samples

Genomic DNA of 33 HLA homozygous cell lines from 15 countries (Additional file 4: Table S3), 11 healthy participants and three families of parents/child trios or quartet were applied. All subjects gave written informed consent for genetic screening. Ethical approvals for this study protocol were obtained from the ethical committees of National Institute of Genetics and Tokai University School of Medicine.

### HLA genotyping with PCR-SSO method

We genotyped *HLA-A*, *HLA-C*, *HLA-B*, *HLA-DRB1*, *HLA-DQB1,* and *HLA-DPB1* using the Luminex assay system and HLA typing kits (WAKFlow HLA Typing kits, Wakunaga, Osaka, Japan or LABType SSO, One Lambda, Canoga Park, CA).

### Sequencing of HLA genes

The six HLA genes (*HLA-A*, *-C*, *-B*, *-DRB1*, *-DQB1,* and *-DPB1*) were each amplified using locus specific primers (Additional file 1: Table S1) with a long-range PCR method. The primers were designed to anneal to conserved regions, i.e., having no recorded variants in dbSNP135. Each amplification reaction contained 20 ng of genomic DNA, 0.25 unit of PrimeSTAR® GXL DNA polymerase (TAKARA BIO INC., Japan), 1× PrimeSTAR® GXL buffer (Mg^2+^ concentration 1 mM), 0.2 mM of each dNTP, and 0.2 μM of each primer in a 10 μl reaction volume. Cycling parameters were as follows: initial denaturation of 94°C/2 min followed by 30 cycles of 98°C/10 sec, 60°C/15 sec and 68°C/10 min. DNA libraries of these PCR products were prepared by a transposase-mediated library preparation method with the Nextera DNA Sample Preparation Kit (Illumina, San Diego, CA, USA) allowing reduced amount of DNA (50 ng) and time for the library construction (90 min). Briefly, PCR product was treated with the Tn5 transposase artificially loaded with synthetic oligonucleotides, enabling simultaneous fragmentation and addition of an adaptor. Each sample was dual indexed and equally pooled by evaluating the molar concentration by Agilent High Sensitivity DNA Kit and 2100 Bioanalyzer (Agilent Technologies, Santa Clara, CA, USA). Fragments ranging from 500 to 2,000 bp (mean size: 1,561 bp), were selected from the pooled library by adding size fractionation step from agarose gel (Figure 1). The library was subjected to multiplex sequencing on the MiSeq sequencer (Illumina). The MiSeq flowcell of 2 × 250 bp paired-end reads resulted in 15 to 17 million read pairs corresponding to 5.8 to 8.5 billion bases of sequence data. The average depths were 157× for homozygous samples with the individual-tagging method and 2,281× for heterozygous samples with the gene-tagging method, respectively. All sequence data associated with this project has been submitted to DDBJ Sequence Reads Archive (DRA accession number DRA000908) and compiled in National Bioscience Database Center (http://biosciencedbc.jp/en/).

### Determination of HLA gene sequences

Sequence reads were distributed according to each index information to assign individuals. Adapter sequence and low quality bases (Phred quality score < Q30) were then excluded and only long (> 200 bp) paired-end reads were selected. These qualified sequence reads were aligned to the 6 HLA gene sequences of UCSC hg19 reference using the BWA (Version 0.5.17; http://bio-bwa.sourceforge.net/) [26] and SAMtools (Version 0.1.18; http://samtools.sourceforge.net/) [27] After the paired-end reads were aligned, SNVs were identified using UnifiedGenotyper in GATK package (http://www.broadinstitute.org/gsa/wiki/index.php/Home_Page) [28,29] and our original perl script to remove false positive SNVs based on the proportion of variant reads to total reads at each SNV site.

In the individual-tagging method for HLA homozygous samples, paired-end reads of the selected size from pooled amplicons were simultaneously aligned to six HLA genes of the hg19 reference sequence, and then six consensus sequences based on the defined SNVs and Indels were obtained. In the gene-tagging method for HLA heterozygous samples, paired-end reads from one amplicon of the HLA gene were aligned to the corresponding HLA gene reference of UCSC hg19 reference, and SNVs and Indels were detected using the same pipeline in the individual-tagging method. Heterozygous SNVs were utilized for HLA gene haplotype phasing. More specifically, paired-end reads showing SNVs in both forward and reverse reads were extracted from the alignment result, and the paired-end reads having heterozygous SNV sites were tiled to generate two HLA gene haplotypes using our original perl script. The script extracts only heterozygous bases from SAM file, and tabulates read-backed HLA gene haplotypes from paired-end reads, and finally tiles subset of HLA gene haplotype to construct complete phased SNVs as HLA gene haplotypes (Figure 2). The two HLA gene haplotype alignments were reconstructed by these phased SNV information from SAM file, and continuous sequences having high consensus quality score (> Q20) [27] were created and searched for databases.

### Search for database and HLA allele determination

The phase-defined HLA gene sequences were subjected to HLA allele determination by searching the IMGT/HLA (http://www.ebi.ac.uk/imgt/hla/) database. Using our method, we were able to obtain complete gene sequences of six HLA genes and it is possible to use those complete gene sequences when searching against the database. However, because most of the sequences in database are only for coding region, we extracted exon sequences and merged them to construct a coding sequence. We then used them as query when doing a BLAT [30] search against known HLA allele sequences in the database, allowing for up to 1% mismatch. The pipeline to construct phased HLA gene sequences, and call HLA alleles up to 8 digit are available in p-galaxy in DDBJ (http://p-galaxy.ddbj.nig.ac.jp) [31], only paired-reads fastq files are required to generate phased HLA gene sequence and determine the allele number.

## Competing interests

The authors declare that they have no competing interests.

## Authors’ contributions

KH and II designed the research and participated in manuscript preparation. KH TAJ and HN developed the bioinformatics method. MS performed HLA typing using SSO method. All authors read and approved the final manuscript.

## Supplementary Material

Additional file 1: Table S1 PCR primers and estimated amplicon size for the 6 HLA genes. Click here for file

Additional file 2: Figure S1 PCR amplification of the six HLA genes. (**A**) Amplified region of the six loci, where dark green boxes represent exons. Red arrows indicate the amplified region. (**B**) Agarose gel electrophoresis of PCR products and sizes of each amplicon. Click here for file

Additional file 3: Table S2 The average frequency of minor allele as an indicator of allelic imbalance in heterozygous samples. Click here for file

Additional file 4: Table S3 The HLA homozygous cell lines and genotype of the HLA loci. Click here for file
