# Supplementary material for: Phase-defined complete sequencing of the HLA genes by next-generation sequencing
Source: BMC Genomics. 2013 May 28;14:355. doi: 10.1186/1471-2164-14-355 (PMC3671147; doi:10.1186/1471-2164-14-355)
Supplement: Additional file 2: Figure S1 — PCR amplification of the six HLA genes. (A) Amplified region of the six loci, where dark green boxes represent exons. Red arrows indicate the amplified region. (B) Agarose gel electrophoresis of PCR products and sizes of each amplicon. [file 1471-2164-14-355-S2.pptx]

## Slide 1
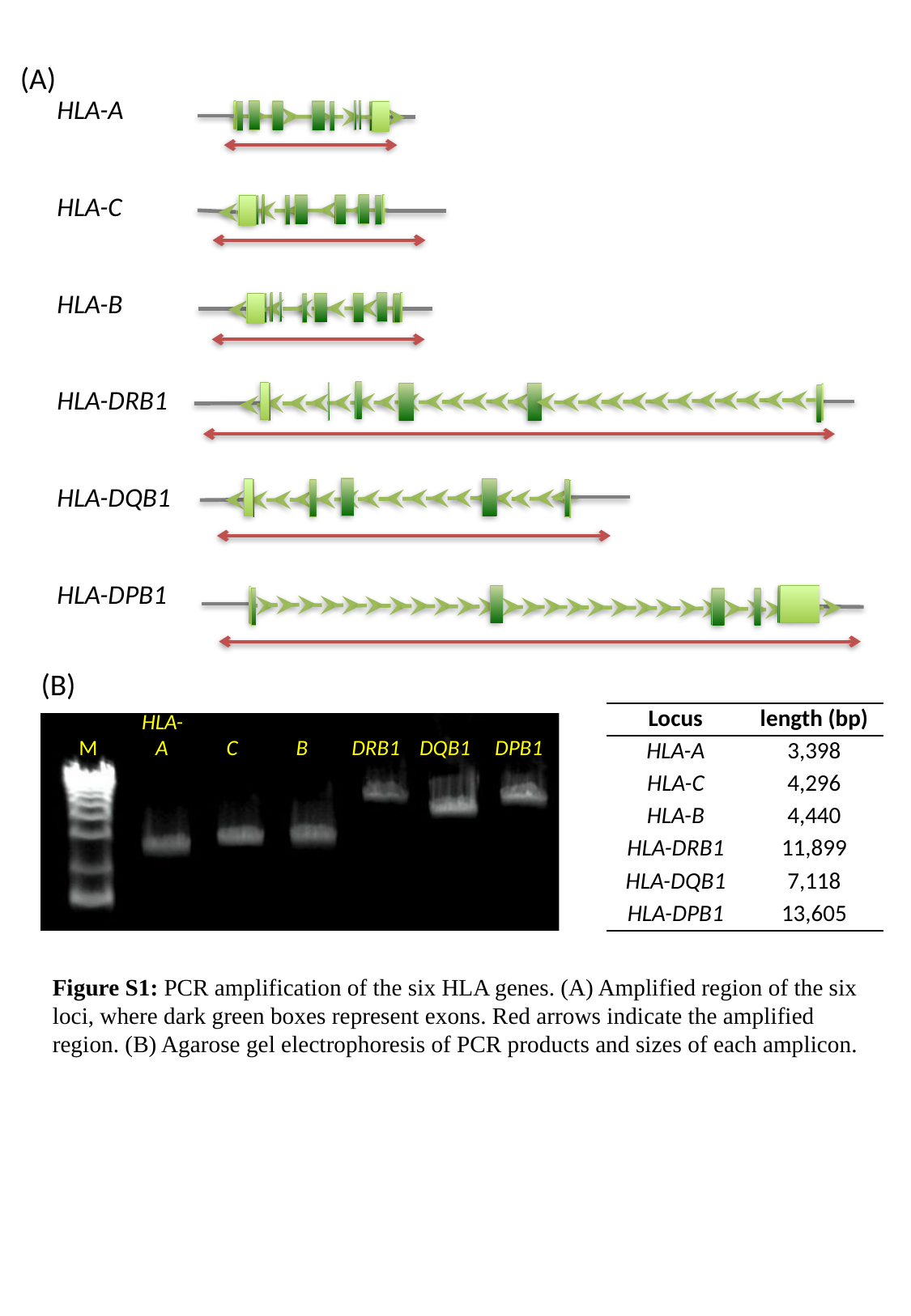

(A)
HLA-A
HLA-C
HLA-B
HLA-DRB1
HLA-DQB1
HLA-DPB1
(B)
 HLA-
 M A C B DRB1 DQB1 DPB1
| Locus | length (bp) |
| --- | --- |
| HLA-A | 3,398 |
| HLA-C | 4,296 |
| HLA-B | 4,440 |
| HLA-DRB1 | 11,899 |
| HLA-DQB1 | 7,118 |
| HLA-DPB1 | 13,605 |
Figure S1: PCR amplification of the six HLA genes. (A) Amplified region of the six loci, where dark green boxes represent exons. Red arrows indicate the amplified region. (B) Agarose gel electrophoresis of PCR products and sizes of each amplicon.
